# Dataset on modelling a synthetic oil rim reservoirs for optimizing oil production during gas cap blow down strategy

**DOI:** 10.1016/j.dib.2022.108652

**Published:** 2022-10-08

**Authors:** Oluwasanmi Olabode, ChukwuEbuka Nnorom, Samuel Ajagunna, Kehinde Awelewa, Precious Umunna, Odera Uzodinma

**Affiliations:** Department of Petroleum Engineering, Covenant University, Ogun State, Nigeria

**Keywords:** Oil rim reservoirs, Gas cap blow down, Reservoir modelling, *K_rw_*, is the water relative permeability, *S_w_*, is the water saturation, *K_ro_*, relative permeability to oil, *S_or_*, residual oil saturation, *S_cw_*, critial oil saturation, FOE, Field Oil Efficiency, FPR, Field Pressure, WOPT, Well Oil Production Total, WWCT, Well Water Cut, WGPT, Well Gas Production Total, OGR, Oil Gas Ratio, HWL, Horizontal Well Length, GOR, Gas Oil Ratio, BHP, Bottom Hole Pressure, Ho, Pay thickness, M-factor, Aqfac, Gas cap and aquifer factors, Ky, Kx, Permeabilities in Y and X direction, Kv/Kh, Anisotropy, BHP, Bottomhole pressure

## Abstract

Oil rim reservoirs with very large gas caps, strong aquifers, and pay thickness below 30 ft. pose oil production challenges to operators. With best operational practices, very high gas oil ratios are recorded at the initial onset of oil production, thus such reservoirs are subjected to a gas cap blow down leading to an ultimate loss in oil reserves. This loss is attributed to a rapid and drastic drop in pressure over the productive life of the reservoir. To maximize oil production, a simulation study is focused on initiating oil wells at different time intervals and estimating oil recoveries at these points. It is believed that the gas cap would have been blown down in time to accommodate for substantial oil production. This study presents the reservoir data (from the Niger-Delta) that can be incorporated in a black oil reservoir simulator (Eclipse) coupled with best production and optimization strategies (water and gas injection) for maximum oil production during gas cap blow down. The data presented will provide a detailed process developing an oil rim synthetic model, support and enhance further studies in optimizing oil production in oil rims subjected to gas cap blow down, create a template for secondary and enhanced oil recovery processes.

**Specification Table**

**Table:** 

Subject	Petroleum Engineering
Specific subject area	Reservoir simulation (Oil production Optimization)
Type of data	Table, Figures, and Graphs
How data were acquired	Obtained from an assessment of oil rim reservoir properties from the Niger-delta region
Data Format	Raw (from oil rim reservoirs), Processed (for grid design)
How data were acquired	Grid data (from mensuration analysis), reservoir rock and fluid properties (design of experiment on oil rim reservoir properties), assumed coordinates for producer and injector wells, suggested injection and production rates from literatures.
Data source location	Department of Petroleum Engineering, Covenant University, Ota, Nigeria
Data Accessibility	1. Data is with article 2. Repository name: Mendeley data DOI:10.17632/gt4gw5hxx7.1 URL: https://data.mendeley.com/datasets/gt4gw5hxx7

## Value of the Data

• The data will introduce various keywords utilized in the Eclipse software used in building a static oil rim model from Niger delta reservoir data. This will also include a mensuration analysis used in the grid design.
• The data will introduce properties such as the PVT, rock and fluid and data such as schedule (for well placement and trajectory) and pressure (under gas cap blow down)
• The dataset provides oil production time intervals suggested for oil production optimization during gas cap blowdown strategy and the suggested injection and production rates applied to maximize oil recovery.

## 1 Data Description

**Fig. 1:**
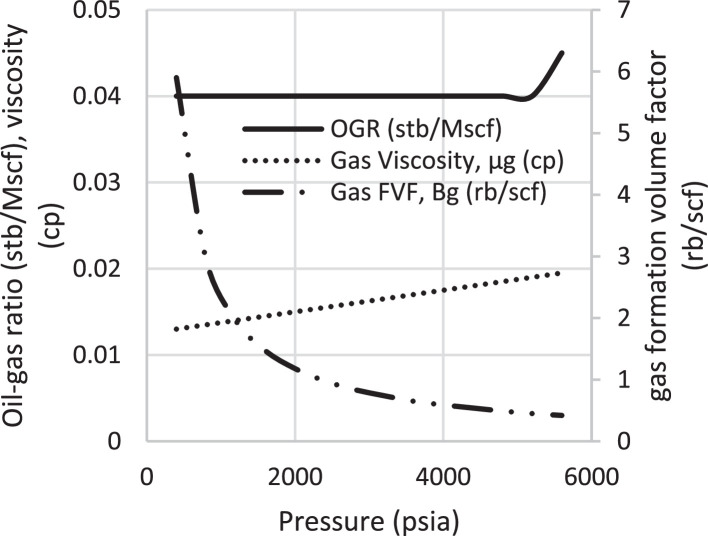
Gas properties.

**Fig. 2:**
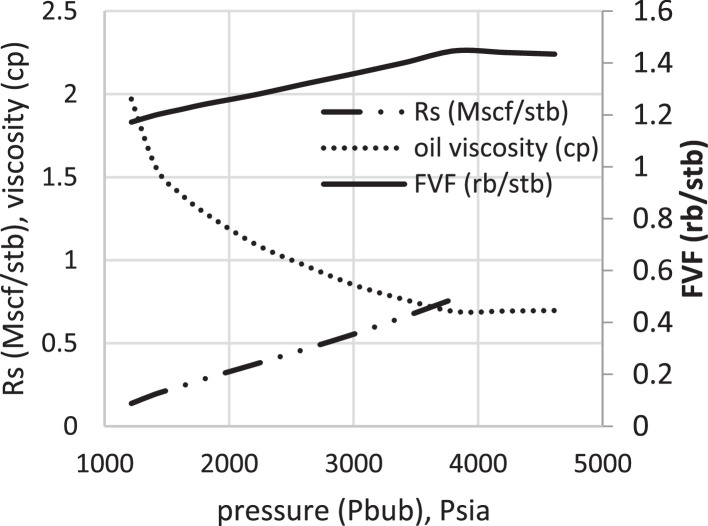
Oil properties.

**Fig. 3:**
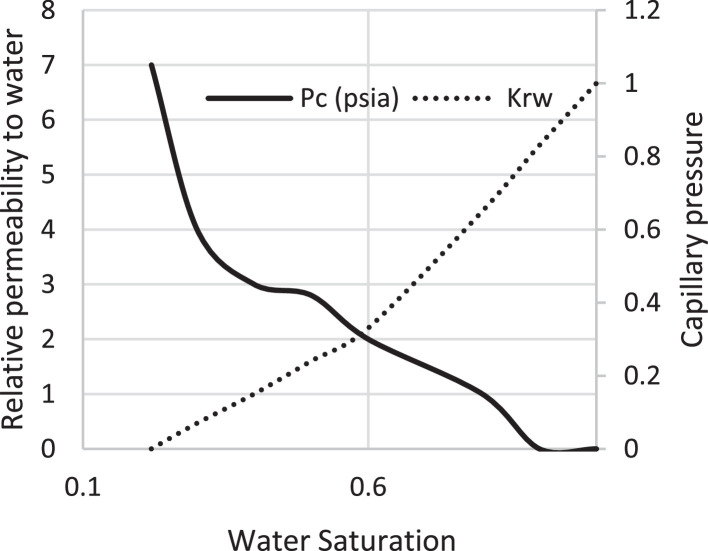
Water saturation.

**Fig. 4:**
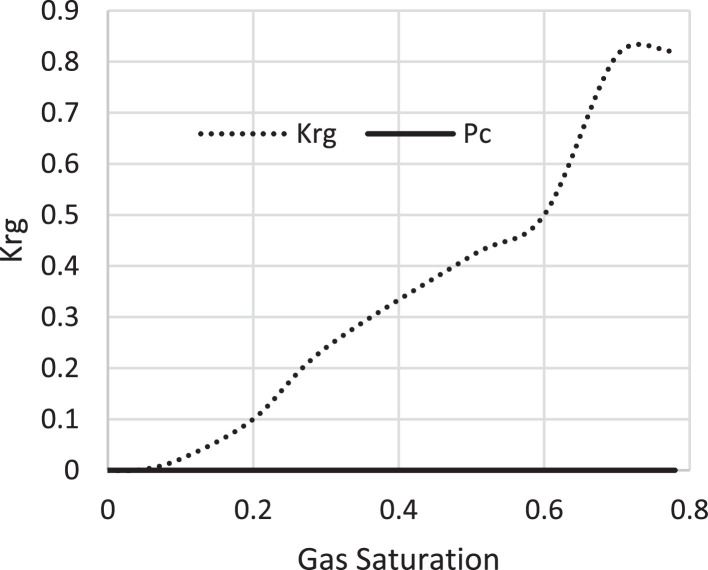
Gas saturation.

**Fig. 5:**
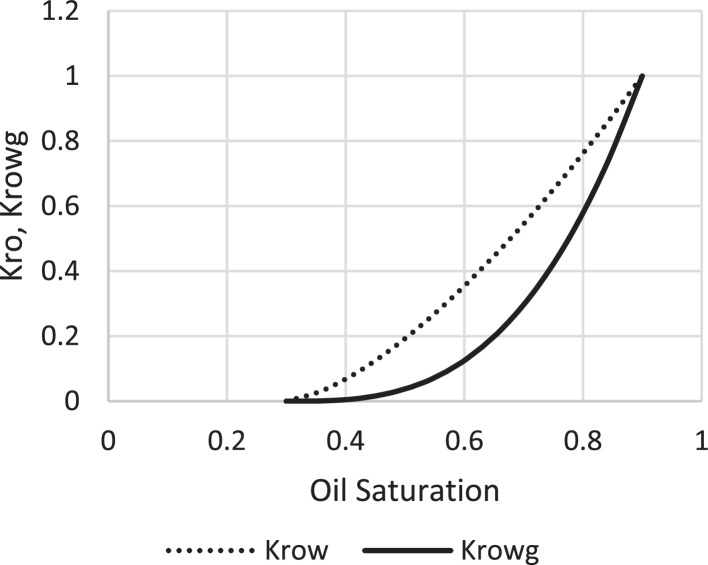
Oil saturation.

Various factors affect the production of oil and gas in oil rim reservoirs asides the production strategies [1,2]. In the absence of real data to represent the oil rim reservoir a static reservoir model can be built using data from more than 50 oil rim reservoir from the Niger delta in conjunction with mensuration analysis using the Eclipse software method. The range of each of these variables has been categorized under low, medium, and high putting the level of occurrence of each value into consideration. A detailed design of experiment has been carried out by [3] using [4] theory on reservoir and operational data from oil rim reservoirs. In this design 15 identified parameters (reservoir and operational) were subjected to a 2-level design of experiment using the Minitab software. A fractional factorial design of experiment has been considered to create subsets of the full factorial design thus taking an advantage of spatial co-effect of variables, reducing computing time, increased resolution, and low-order interaction of variables. The operation is designed to create subject models with different uncertainty values which are inputted into the Eclipse software in conjunction with PVT, Solution, Rock and fluid properties to create oil rim models. This theory is used to create 18 base study models of oil rim reservoirs which after proper classification with respect to the pay thickness, and sizes/indices of the gas cap and aquifer can be subjected to the 4 production strategies (Concurrent, Swing, Sequential, and Gas cap blowdown) proposed by [5,6]. Table 1 shows the outcome of the base case distribution of uncertainties using the design of experiment with the definition of these parameters shown in the appendix section. Models D, K, AND L easily fits into a model that can be subjected to a gas-cap blow down strategy considering the values of the gas-cap, pay thickness and aquifer. To construct the initial design of model, the dip angle of the reservoir is taken into consideration. The base assumption is that the reservoir is a box in sitting laterally on a horizontal plane with 3 dimensional coordinates of x, y and z. A keyword TOPS (in the grid section) is used to indicate the depth from the surface to top of the reservoir. The Tops is subsequently used to populate the degree of deviations in various cells in the x, y, and z directions considering the number of cells in each direction (i.e., 20 by 25 by 20), the dip angle, magnitude of the gas-cap and aquifer sizes. The outcome of the TOPS is described in Table 3 and further details and steps have been fully captured by [7]. At the grid section, the keyword PORO is used to input the porosity values for 10,000 cells Table 2) where each respective 500 cells have different porosity values. Taking model K as an example, the permeability is the x and y direction is 3500 md, while that in the z direction is 35 md (as indicated by the fraction in column 8). Oil, water, gas, dissolved gas and vaporized oil properties are indicated at the RUNSPEC section while the PVT section analyses the properties of these reservoir fluids in conjunction properties. For example, the property of gas with vaporized oil is indicated by the keyword PVDG (Fig. 1) and it shows the relationship between the Gas pressures with respect to the oil gas ratio and gas formation volume factor while the oil property (with dissolved gas) is denoted by the keyword PVTO (Fig. 2) describing the relationship between the bubble point pressure with respect to the solution gas and capillary pressure. The Corey equation is used to estimate the oil and water saturation end points with their respective relative permeabilities are expressed in Eqs. (1) and ((2) and resulting plots in Figs. 3, 4, and 5 respectively. A reference pressure of 4000 psia is initiated as the rock pressure, water formation volume factor of 1.0043 rb/stb, water viscosity of 0.513 cp, water viscosibility of 0, with water and rock compressibility factors of 3 × 10^−6^ and 4.2 × 10^−6^ respectively.

(1) $$K_{rw}\left(S_{w}\right)=K_{rw,or}{\left(\frac{S_{w}-S_{cw}}{1-S_{cw}-S_{or}}\right)}^{nw}$$

(2) $$K_{ro}\left(S_{w}\right)=K_{rw,cw}{\left(\frac{1-S_{w}-S_{or}}{1-S_{cw}-S_{or}}\right)}^{nw}$$

**Table 1:** Base case models.

Model	Dip	OGR	Ho (ft.)	m-Factor	Aqfac	Kx, Ky	Kv/Kh	Bore Diam. (ft)	OIL DENSITY	HGOC (ft.)	HWL (ft.)	Qo	Krw	GOR (*Rsi)	BHP (psia)
**A**	6	0.04	70	6	6	3500	0.1	0.55	47	0.6	1800	3500	0.6	7.5	2200
**B**	1	0.04	20	6	0.7	3500	0.01	0.55	37	0.6	1200	3500	0.2	7.5	1500
**C**	6	0.006	20	6	6	35	0.01	0.55	47	0.25	1200	3500	0.6	2.5	1500
**D**	1	0.006	70	6	0.7	35	0.1	0.55	37	0.25	1800	3500	0.2	2.5	2200
**E**	6	0.04	70	0.7	0.7	35	0.01	0.55	47	0.6	1800	1200	0.2	2.5	1500
**F**	1	0.04	20	0.7	6	35	0.1	0.55	37	0.6	1200	1200	0.6	2.5	2200
**G**	6	0.006	20	0.7	0.7	3500	0.1	0.55	47	0.25	1200	1200	0.2	7.5	2200
**H**	1	0.006	70	0.7	6	3500	0.001	0.55	37	0.25	1800	1200	0.6	7.5	1500
**I**	6	0.04	70	6	6	3500	0.1	0.35	37	0.25	1200	1200	0.2	2.5	1500
**J**	1	0.04	20	6	0.7	3500	0.01	0.35	47	0.25	1800	1200	0.6	2.5	2200
**K**	6	0.006	20	6	6	35	0.01	0.35	37	0.6	1800	1200	0.2	7.5	2200
**L**	1	0.006	70	6	0.7	35	0.1	0.35	47	0.6	1200	1200	0.6	7.5	1500
**M**	6	0.04	70	0.7	0.7	35	0.01	0.35	37	0.25	1200	3500	0.6	7.5	2200
**N**	1	0.04	20	0.7	6	35	0.1	0.35	47	0.25	1800	3500	0.2	7.5	1500
**O**	6	0.006	20	0.7	0.7	3500	0.1	0.35	37	0.6	1800	3500	0.6	2.5	1500
**P**	1	0.006	70	0.7	6	3500	0.01	0.35	47	0.6	1200	3500	0.2	2.5	2200
**Q**	1	0.006	20	0.7	0.7	35	0.01	0.35	37	0.25	1200	1200	0.2	2.5	1500
**R**	4	0.03	40	3	3	350	0.01	0.45	42	0.45	1500	2200	0.35	5	1800

**Table 2:** Porosity values.

500×0.29	500×0.24	500×0.27	500×0.26	500×0.28	500×0.25	500×0.26	500×0.28	500×0.26	500×0.28	500×0.29
500×0.24	500×0.25	500×0.24	500×0.27	500×0.28	500×0.29	500×0.28	500×0.29	500×0.00

**Fig. 6:**
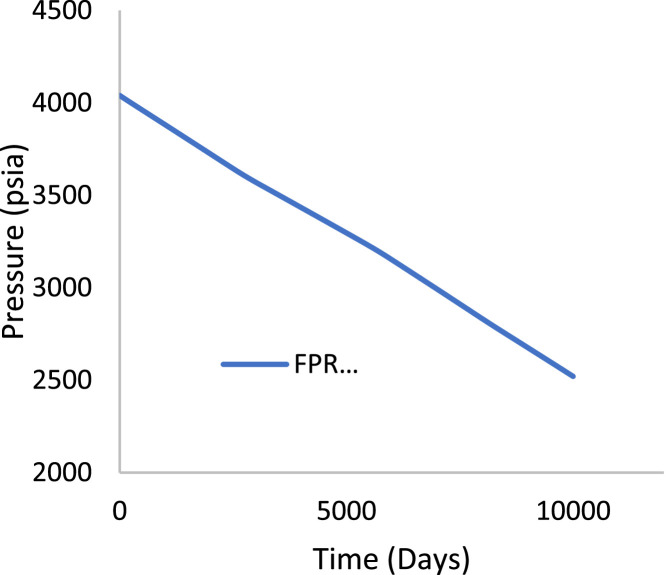
Pressure decline.

**Fig. 7:**
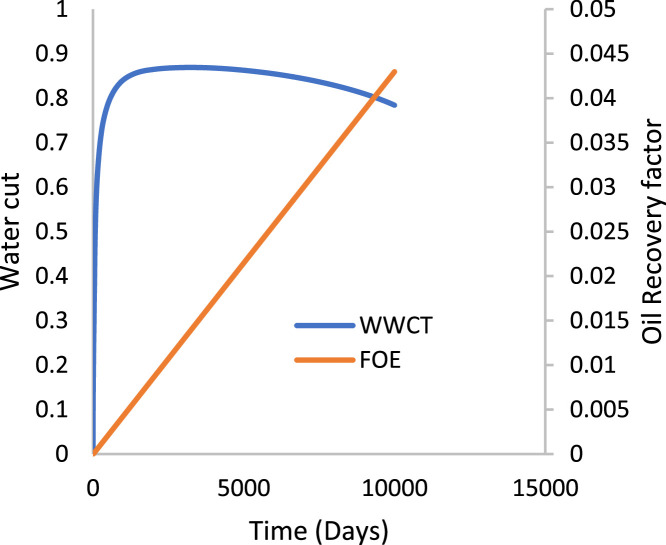
Oil recovery & water cut.

**Fig. 8:**
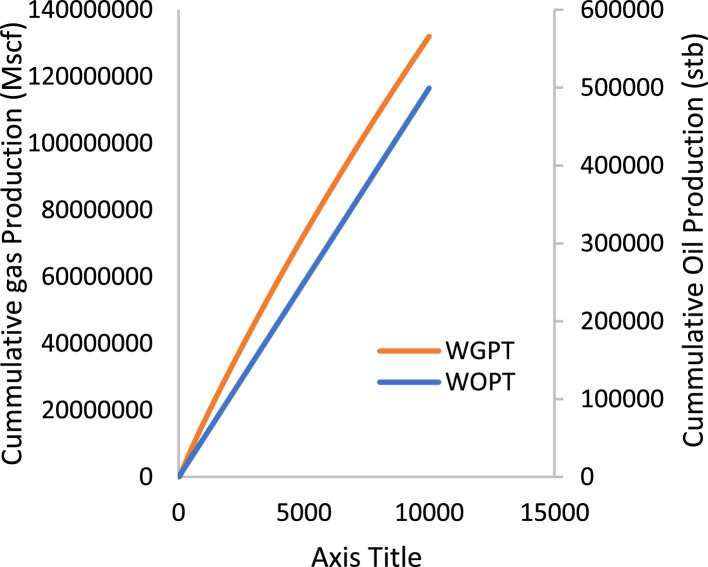
Cumulative oil and gas production.

**Fig. 9:**
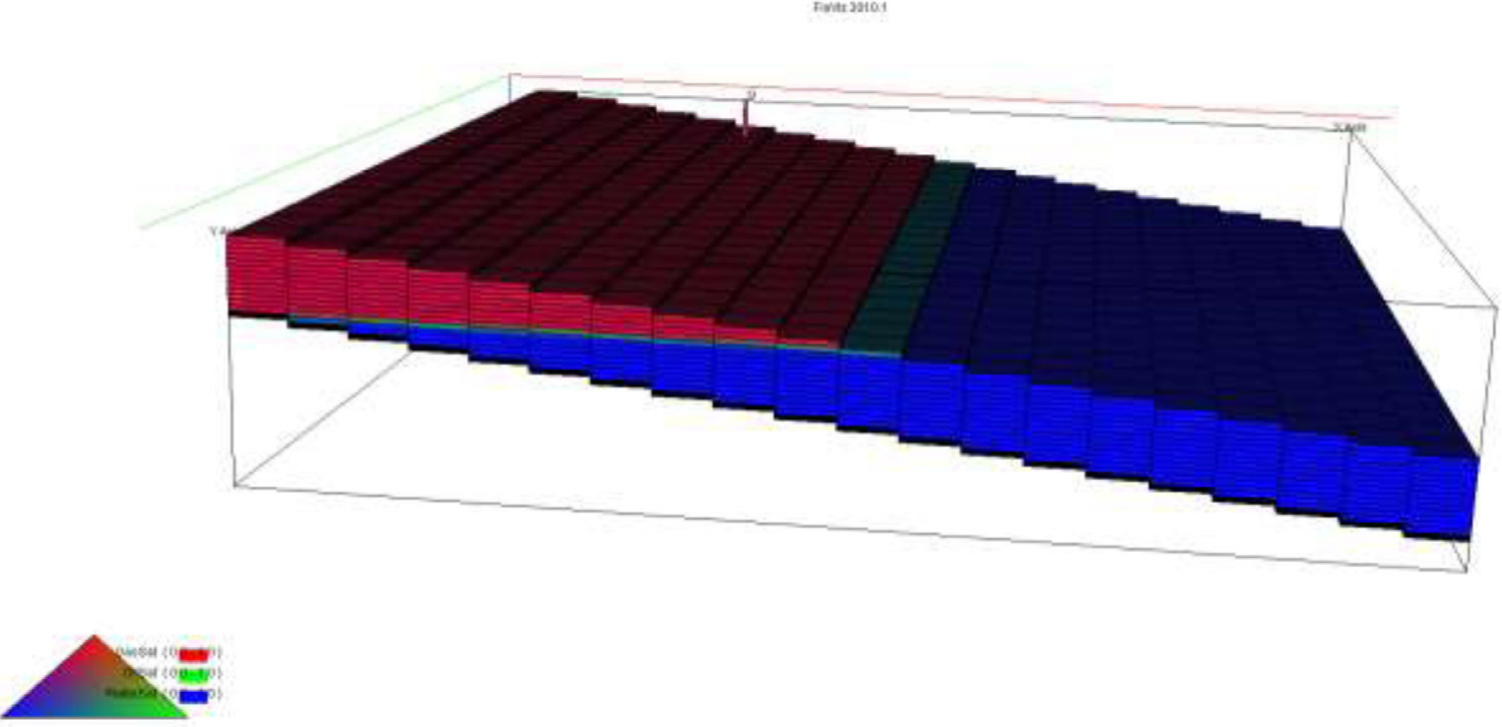
Ternary diagram of fluid distribution.

The initial fluid dimension placements in the reservoir have been described by the keyword EQUALS (Table 3) where the sizes of each cell in the x, y and z directions are 300 ft., 300 ft., and 30 ft. respectively. Table 3 describes the allocation of fluid properties per depth of the reservoir. The red, green, and blue colors represent the gas, oil, and water regions respectively. The equilibration data which specifies the fluid contact (water oil and gas oil contacts) at a specific datum depth and pressure (i.e., 7000 ft. and 4000 psia respectively) is indicated with the key word EQUIL under the initialization section. The initialization of the reservoir resulted in the reserves estimate in Table 4 and a ternary diagram (Fig. 9) showing the reservoir fluid placement and description. The concept of well placements in oil rim reservoirs proposed by [7] and this can be applied under this strategy of optimizing oil production under gas cap blow down strategy. A total time of 10,000 days (27.4 years) is created at the Schedule section with a time step of 30 days. A gas well is initiated for the gas cap blow down at the start of simulation and the pressure decline is shown in Fig. 6. An oil recovery factor of 4.3% (Fig. 7) is recorded during this strategy at an estimated oil volume of 500,000 stb (Fig. 8). Figs. 1 to 5 are the various oil, water and gas properties inputted into different sections of the software to necessitate the estimation of original fluids in place (Table 4). The property of the gas is with vaporized oil (hence the oil gas ratio function) while the oil property is with dissolved gas hence the oil gas ratio property.

**Table 3:** TOPS data at dip angle of 6°.

8500	8522	8544	8566	8588	8610	8632	8654	8676	8698	8720	8742	8764	8786	8808	8830	8852	8874	8896	8918	8940
8500	8522	8544	8566	8588	8610	8632	8654	8676	8698	8720	8742	8764	8786	8808	8830	8852	8874	8896	8918	8940
8500	8522	8544	8566	8588	8610	8632	8654	8676	8698	8720	8742	8764	8786	8808	8830	8852	8874	8896	8918	8940
8500	8522	8544	8566	8588	8610	8632	8654	8676	8698	8720	8742	8764	8786	8808	8830	8852	8874	8896	8918	8940
8500	8522	8544	8566	8588	8610	8632	8654	8676	8698	8720	8742	8764	8786	8808	8830	8852	8874	8896	8918	8940
8500	8522	8544	8566	8588	8610	8632	8654	8676	8698	8720	8742	8764	8786	8808	8830	8852	8874	8896	8918	8940
8500	8522	8544	8566	8588	8610	8632	8654	8676	8698	8720	8742	8764	8786	8808	8830	8852	8874	8896	8918	8940
8500	8522	8544	8566	8588	8610	8632	8654	8676	8698	8720	8742	8764	8786	8808	8830	8852	8874	8896	8918	8940
8500	8522	8544	8566	8588	8610	8632	8654	8676	8698	8720	8742	8764	8786	8808	8830	8852	8874	8896	8918	8940
8500	8522	8544	8566	8588	8610	8632	8654	8676	8698	8720	8742	8764	8786	8808	8830	8852	8874	8896	8918	8940
8500	8522	8544	8566	8588	8610	8632	8654	8676	8698	8720	8742	8764	8786	8808	8830	8852	8874	8896	8918	8940
8500	8522	8544	8566	8588	8610	8632	8654	8676	8698	8720	8742	8764	8786	8808	8830	8852	8874	8896	8918	8940
8500	8522	8544	8566	8588	8610	8632	8654	8676	8698	8720	8742	8764	8786	8808	8830	8852	8874	8896	8918	8940
8500	8522	8544	8566	8588	8610	8632	8654	8676	8698	8720	8742	8764	8786	8808	8830	8852	8874	8896	8918	8940
8500	8522	8544	8566	8588	8610	8632	8654	8676	8698	8720	8742	8764	8786	8808	8830	8852	8874	8896	8918	8940
8500	8522	8544	8566	8588	8610	8632	8654	8676	8698	8720	8742	8764	8786	8808	8830	8852	8874	8896	8918	8940
8500	8522	8544	8566	8588	8610	8632	8654	8676	8698	8720	8742	8764	8786	8808	8830	8852	8874	8896	8918	8940
8500	8522	8544	8566	8588	8610	8632	8654	8676	8698	8720	8742	8764	8786	8808	8830	8852	8874	8896	8918	8940
8500	8522	8544	8566	8588	8610	8632	8654	8676	8698	8720	8742	8764	8786	8808	8830	8852	8874	8896	8918	8940
8500	8522	8544	8566	8588	8610	8632	8654	8676	8698	8720	8742	8764	8786	8808	8830	8852	8874	8896	8918	8940

**Table 4:** Reserves estimates.

Oil (stb)		Water (stb)	Gas (Mscf)	
Liquid	Vapour	0	Free	Dissolved
22,939,991	2558,384	0	426,397,378	19,298,821
25,498,376		559,407,219	445,696,199

## 2 Experimental Design, Materials and Methods

Oluwasanmi [8] has highlighted 4 production strategies for oil rim reservoirs with respect to the pay thickness, gas cap and aquifer sizes. To create synthetic models of oil rim reservoirs that will befit a gas cap blow down strategy, a 2-level Placket Burman design of experiment is utilized using the Minitab software [1]. Data is gathered on factors that affect oil productivity in oil rim reservoirs (first column of Table 1) and used to generate 18 different oil rim models. From Table 1, models C, J and K fit a description of those to undergo a gas cap blow down strategy with pay thickness less than 20 ft. and a sizably large gas cap. These models can easily be classified as ultra-thin reservoir models with large gas cap and aquifers (models C and K) and large gas cap and small aquifer (model J) [8]. To accommodate the dip angle reservoir property mensuration analysis developed by [3]. The report from their work can be useful to generate a TOPS (Table 2) database (using the angle of dip, assumed heights of the gas cap and aquifers based on their indexes in Table 1 and the pay thickness) to be uploaded in the grid section of the Eclipse software.

Two horizontal wells are completed in the gas cap (for gas production) and mid-stream (for oil production). Oil and gas production rates of 1500 stb/day and 10,000 mscf/day has been selected as proposed by [9], [10], [11], [12] has proposed a simultaneous/smart production method from a single well to reduce costs incurred in drilling an extra well. The rapid decline in reservoir pressure has resulted in the low oil recovery factor experienced hence a need to optimize the production of oil. The usual practice in oil rim reservoirs is to optimize oil production in such a way that production of gas is not jeopardized, especially when there is a market for gas. For oil rims of the nature described under gas cap blow down, the onset of oil production will lead to high gas oil ratios resulting in some loss in capital expended on oil production. Hence from the pressure profile, it is expected that the reservoir pressure will decline to a point along the time interval of 10,000 days that will accommodate for maximum exploitation of oil and possible additional increase via secondary injection schemes at those time intervals. Thus, oil production is initiated at 2000, 4000, 6000 and 8000 days ascertain the level of oil recovery and compare them with that recovered during gas cap blow down. The well description data is described in Table 6 and to maximize oil recovery via secondary injection means, sensitivity analysis on different injection rates described in Table 5 is initiated. This description can be implemented with enhanced oil recovery [14] and injection pattern strategies for oil rim reservoirs [13]. Each well description is individually applied to each time step and the simulation results (oil recovery, oil and gas production) are respectively estimated and compared for each time step (Table 7).

**Table 5:** Pressure management strategy.

Water injection (stb/day)			Gas injection (Mscf/day)		
1000	2000	3000	10,000	20,000	30,000

**Table 6:** Schedule description of producer and injector well.

DAYS/WELL	WELL G	WELL O	WELL BASSY	WELL PELU-INJ
**TIME STEP-10days**				
[0] 01 JANUARY 2022	WELSPEC	SHUT	SHUT	WELSPEC
	Group-G1			Group-G1
	location (I,j)- (6,2)			location (I,j)- (7,13)
	Preferred phase-gas			Preferred phase-gas
	ASI- shut			ASI- shut
	COMPDAT			COMPDAT
	WBID. −0.333ft			WBID. −0.5ft
	Direction- X			Direction- Z
	Has 3-completions			Has 3-completions
	(6,2)			(7,13)
	(Ku, Kl) -(1,1); OPEN			(Ku, Kl) -(1,1); OPEN
	(Ku, Kl) -(2,2); OPEN			(Ku, Kl) -(2,2); OPEN
	(Ku, Kl) -(3,3); OPEN			(Ku, Kl) -(3,3); OPEN
	WCONPROD			WCONINJE
	OPEN			OPEN
	rg-10,000 Mscf/day			rsl-10,000 stb/day
	bhpwell-1500bbls/d			rsg-10,000 Mscf/day

[2000] 24 JUNE 2027	SHUT	WELSPEC	WELSPEC	SHUT
		Group-G1	Group-Producer	
		location (I,j)- (1,19)	location (I,j)- (11,14)	
		Preferred phase-oil	Preferred phase-oil	
		ASI- shut	ASI- shut	
		COMPDAT	COMPDAT	
		WBID. −0.333ft	WBID. −0.5ft	
		Direction- Y	Direction- Z	
		Has 5-completions	Has 5-completions	
		(Ku, Kl) -(14,14); @ (1,19**)** OPEN	(Ku, Kl) -(1,1); @ (11,14**)** OPEN	
		(Ku, Kl) -(15,15); @ (1,18) OPEN	(Ku, Kl) -(2,2); @ (10,14) OPEN	
		(Ku, Kl) -(16,16); @ (1,17) OPEN	(Ku, Kl) -(3,3); @ (9,14) OPEN	
		(Ku, Kl) -(17,17); @ (1,16) OPEN	(Ku, Kl) -(4,4); @ (8,14) OPEN	
		(Ku, Kl) -(18,18); @ (1,15) OPEN	(Ku, Kl) -(5,5); @ (7,14) OPEN	
		WCONPROD	WCONPROD	
		OPEN	OPEN	
		ro-3000 stb/day	ro-2500 stb/day	
		WECON		
		Minimum oil rate- 0.9 stb/day		
		Minimum gas rate- 12.6 Mscf/day		
		Field gas production rate-5000Mscf		
		Maximum water cut limit −0.90		
		Maximum gas-oil ratio −12.6 Mscf/stb		
		Maximum gas-liquid ratio of 150 Mscf/stb		
[4000] 14 DECEMBER 2032	OPEN	SHUT	SHUT	NIL
			
[6000] 06 JUNE 2038	SHUT	OPEN	OPEN	NIL
			
[8000] 27 NOVEMBER 2043	OPEN	SHUT	SHUT	NIL
			
[10,000] 19 MAY 2049	SHUT	SHUT	SHUT	SHUT

**Table 7:** Fluids equilibrium description.

'DX' 200 /
'DY' 200 /
'DZ' 20 1 20 1 20 1 1 /
'DZ' 20 1 20 1 20 2 2 /
'DZ' 20 1 20 1 20 3 3 /
'DZ' 20 1 20 1 20 4 4 /
'DZ' 20 1 20 1 20 5 5 /
'DZ' 20 1 20 1 20 6 6 /
'DZ' 20 1 20 1 20 7 7 /
'DZ' 20 1 20 1 20 8 8 /
'DZ' 13 1 20 1 20 9 9 /
'DZ' 10 1 20 1 20 10 10 /
'DZ' 20 1 20 1 20 11 11 /
'DZ' 20 1 20 1 20 12 12 /
'DZ' 20 1 20 1 20 13 13 /
'DZ' 20 1 20 1 20 14 14 /
'DZ' 20 1 20 1 20 15 15 /
'DZ' 20 1 20 1 20 16 16 /
'DZ' 20 1 20 1 20 17 17 /
'DZ' 20 1 20 1 20 18 18 /
'DZ' 20 1 20 1 20 19 19 /
'DZ' 201 20 1 20 20 20 /

## Ethics Statement

The data presented does not involve any experimentations on humans or animals.

## CRediT authorship contribution statement

**Oluwasanmi Olabode:** Methodology, Conceptualization. **ChukwuEbuka Nnorom:** Writing – review & editing, Supervision. **Samuel Ajagunna:** Writing – review & editing, Writing – original draft. **Kehinde Awelewa:** Writing – review & editing, Supervision. **Precious Umunna:** Writing – original draft. **Odera Uzodinma:** Supervision, Methodology.

## Declaration of Competing Interest

There are no known interests (both financial and individual) which can be perceived to have an influence on this research work.

## Data Availability

Data set on Well trajectory, grid design and oil rim reservoir properties (Original data) (Mendeley Data).
